# Supplementary material for: Endogenous fluctuations of OCT4 and SOX2 bias pluripotent cell fate decisions
Source: Mol Syst Biol. 2019 Sep 25;15(9):e9002. doi: 10.15252/msb.20199002 (PMC6759502; doi:10.15252/msb.20199002)
Supplement: Supplementary file 1 — Expanded View Figures PDF [file MSB-15-e9002-s001.pdf]

Expanded View Figures

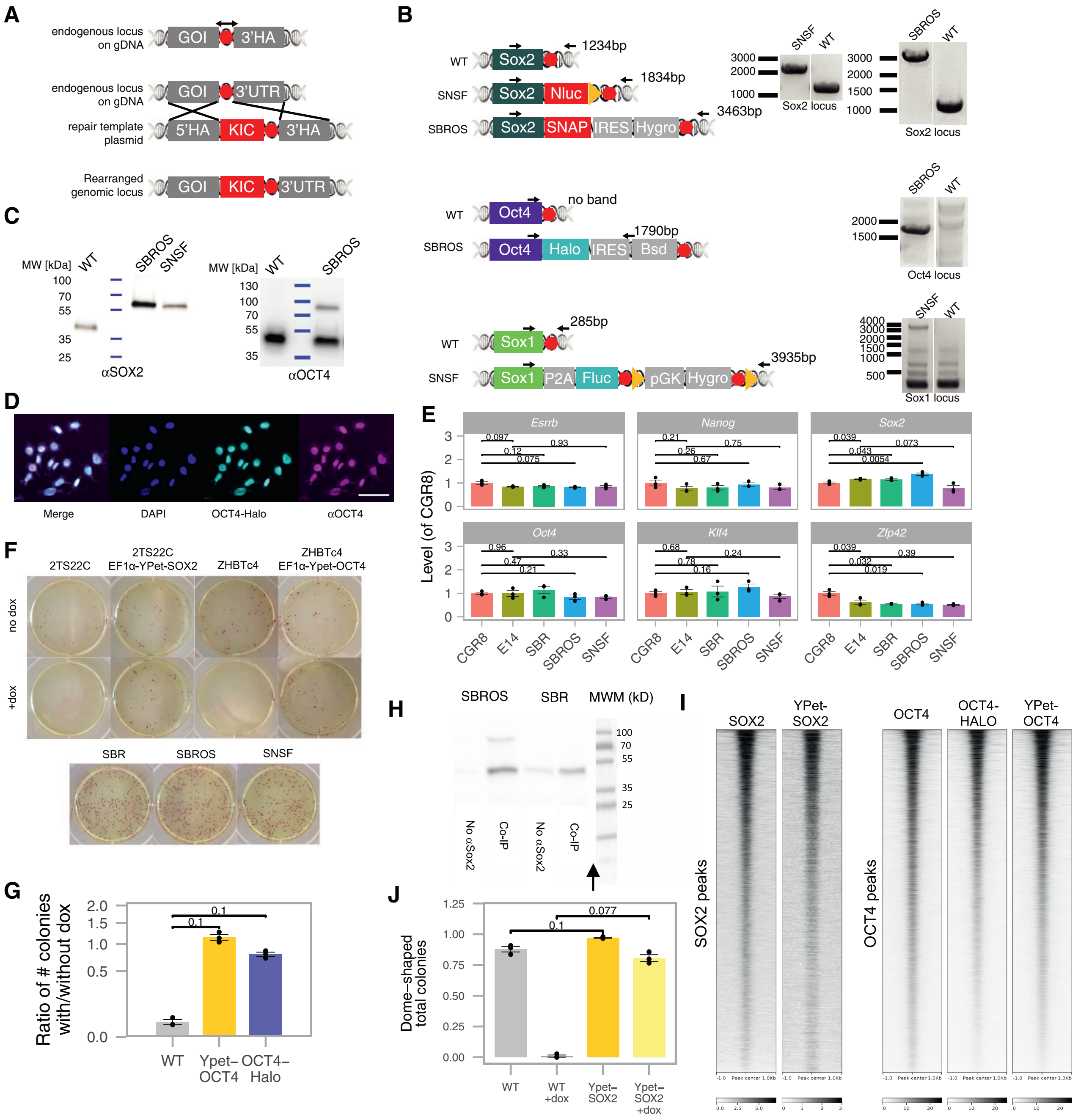

Figure EV1.

© 2019 The Authors

Figure EV1. Validation of knock-in cell lines.

A Schematic of the general knock-in strategy used in this study. Red hexagons: STOP codons; KIC: knock-in construct.
B Location of primers (arrows) and PCR results to verify knocked-in reporters in the SNSF and SBROS cell lines. Red hexagon: STOP codons; Yellow triangles: LoxP sites.
C Western blot confirming the tagging of endogenous SOX2 and OCT4 in the SNSF and SBROS cell lines.
D Example of field of view used for the correlation analysis of total OCT4 (αOCT4) and heterozygous OCT4-HALO. Scale bar: 50 μm.
E qPCR analysis of pluripotency marker expression for cell lines used in this study, normalized to Rps9 and to the levels in CGR8 WT mouse ES cells (n = 3). P-values: two-sided t-test with unequal variance. Error bars: SE.
F Representative images of alkaline phosphatase staining in the different cell lines.
G Ratio of the number of colonies with and without dox, for WT, YPet-OCT4 or OCT4-HALO-expressing Zhbtc4 cells (n = 3). P-values: Mann–Whitney U-test. Error bars: SE.
H Co-immunoprecipitation (Co-IP) of OCT4 and OCT4-HALO with SOX2-SNAP (SBROS) and OCT4 with WT SOX2 (SBR). Arrow: Samples and molecular weight marker (MWM) were spliced together.
I Heatmaps for SOX2 (GSE89599) and YPet-SOX2 ChIP-seq signals in SOX2-bound regions and OCT4 (in SBR background), OCT4-HALO and YPet-OCT4 ChIP-seq signals in OCT4-bound regions.
J Ratio of dome-shaped/flat colonies for WT or YPet-SOX2-expressing 2TS22C cells in the absence or presence of dox (n = 3). P-value: Mann–Whitney U-test. Error bars: SE.

Figure EV2. Cross-regulation of SOX2 and OCT4.

A–C YPet-OCT4 overexpression time-course. A Fluorescence microscopy images after immunofluorescence staining of SOX2 and OCT4. OE: overexpressed YPet-OCT4. B-C Quantifications of total SOX2 and OCT4 levels in single cells upon YPet-OCT4 overexpression, normalized to the average at 0 h. Whiskers: minimum and maximum values; Box: lower and upper quartiles; Solid line: median; solid points: outliers.
D–F YPet-SOX2 overexpression time-course. D Fluorescence microscopy images after immunofluorescence staining of SOX2 and OCT4. OE: overexpressed YPet-SOX2. E-F Quantifications of total SOX2 and OCT4 levels in single cells upon YPet-SOX2 overexpression, normalized to the average at 0 h. Whiskers: minimum and maximum values; Box: lower and upper quartiles; Solid line: median; solid points: outliers.
G Total OCT4 or SOX2 levels as a function of YPet-OCT4 or YPet-SOX2 overexpression, respectively, 4 h (blue) and 7 h (red) after addition of dox.
H Schematic of the knock-in alleles in the SNSF cell line (Red hexagons: STOP codons; Yellow triangles: LoxP sites) and luminescence microscopy images of differentiating SNSF cells showing the SOX2-NLUC and SOX1-P2A-FLUC signal. Scale bars: 50 μm.
I Distributions of OCT4 (n = 2682), SOX2 (n = 1236) and NANOG (n = 2416) levels in WT E14 and SNSF cell lines as determined by quantitative immunofluorescence. Dashed lines: mean protein levels; Dotted lines: median protein levels.
J Cell cycle duration of E14 WT (n = 6) and SNSF (n = 6) cells. Error bars: SE. P-value: two-sided t-test with unequal variance.
K Correlation between SOX2-SNAP initial fluorescence intensity and OCT4-HALO half-life in single cells induced for SOX2-SNAP overexpression (red) or not (black); values are normalized on the average OCT4-HALO half-life and SOX2-SNAP intensity of the no dox condition. P-value is based on Pearson correlation. R is Pearson's correlation coefficient.
L Strategy to sort G1 cells for medium endogenous levels of SOX2 and high or low OCT4 levels (top), and conversely (bottom).
M Raw flow cytometry data of Hoechst profile 8 h after sorting as indicated in panel (L).

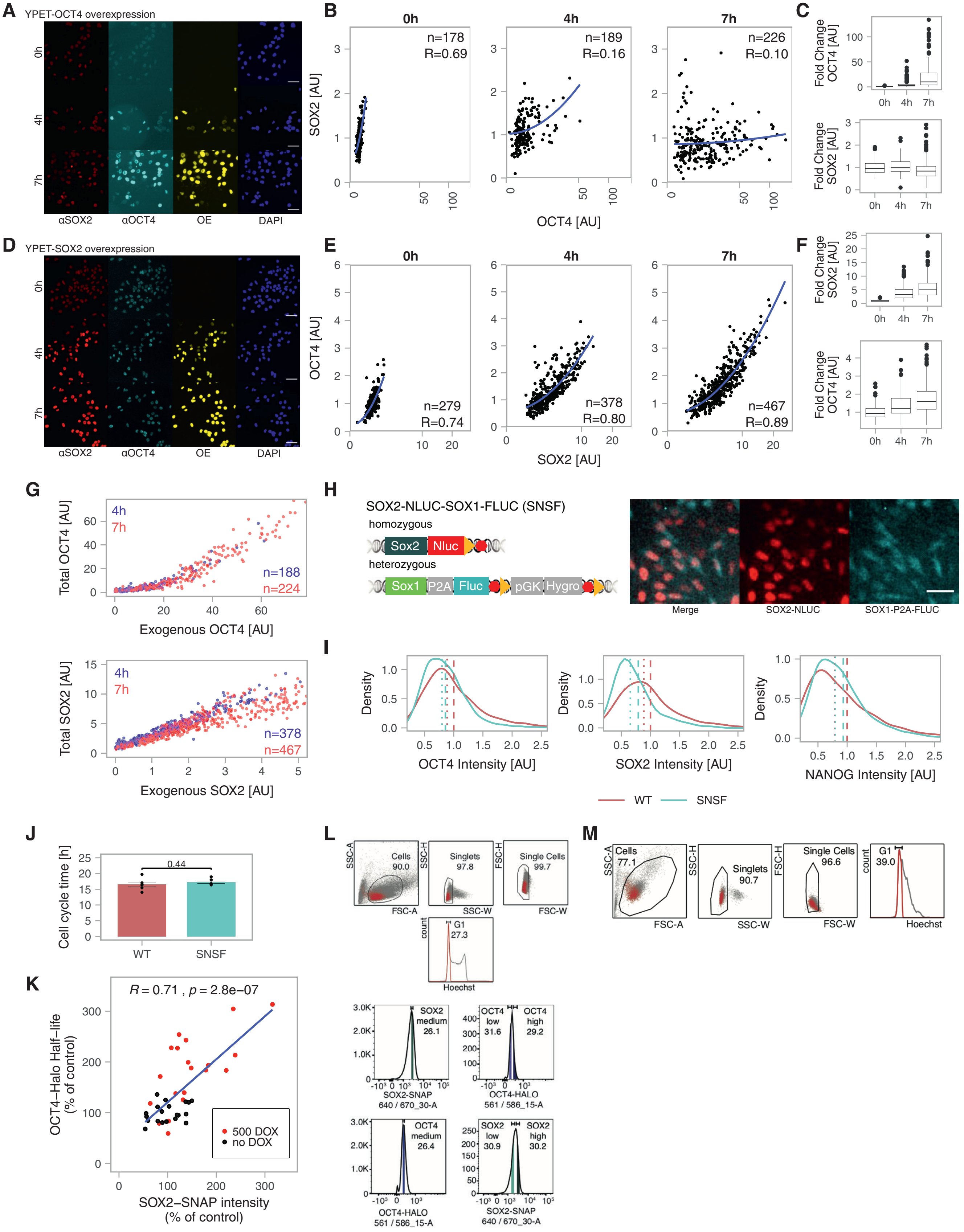

Figure EV2.

 © 2019 The Authors

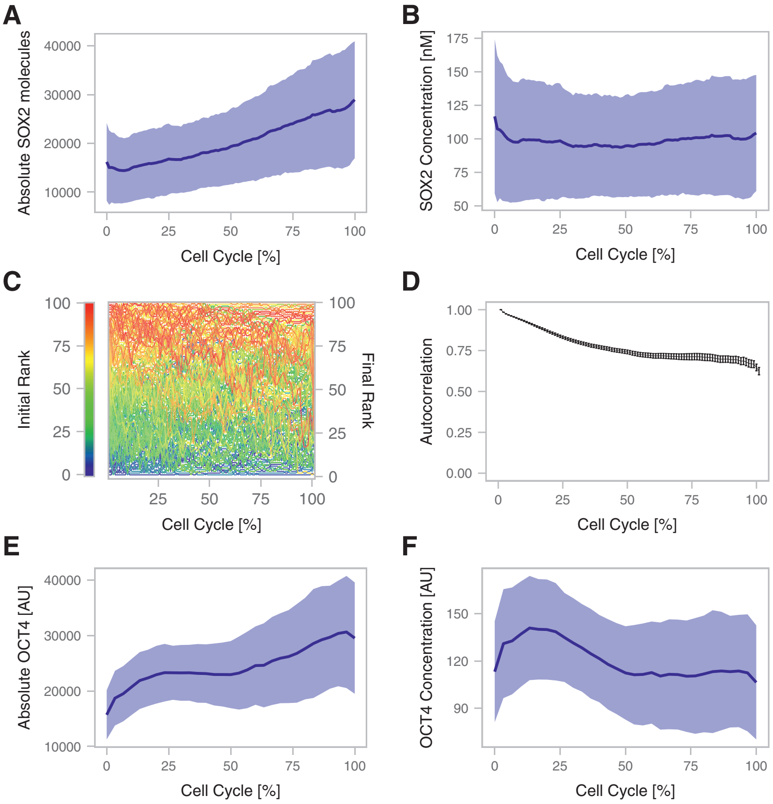

Figure EV3. Fluctuations of SOX2 and OCT4 over the cell cycle.

A, B Average SOX2 protein level (A) and concentration (B) in SNSF cells (n = 164). Shaded area: SD; solid lines: mean.

C Changes of single-cell ranks of SOX2 levels over time using data from one cell cycle (n = 100). Red: initially high expressing cells; Blue: initially low expressing cells.

D Rank-based autocorrelation function of the SOX2 ranks. Error bars: SE estimated by bootstrapping.

E, F Average OCT4 protein level (E) and concentration (F) (n = 48) in SBROS cells. Shaded area: SD; solid lines: mean.

© 2019 The Authors

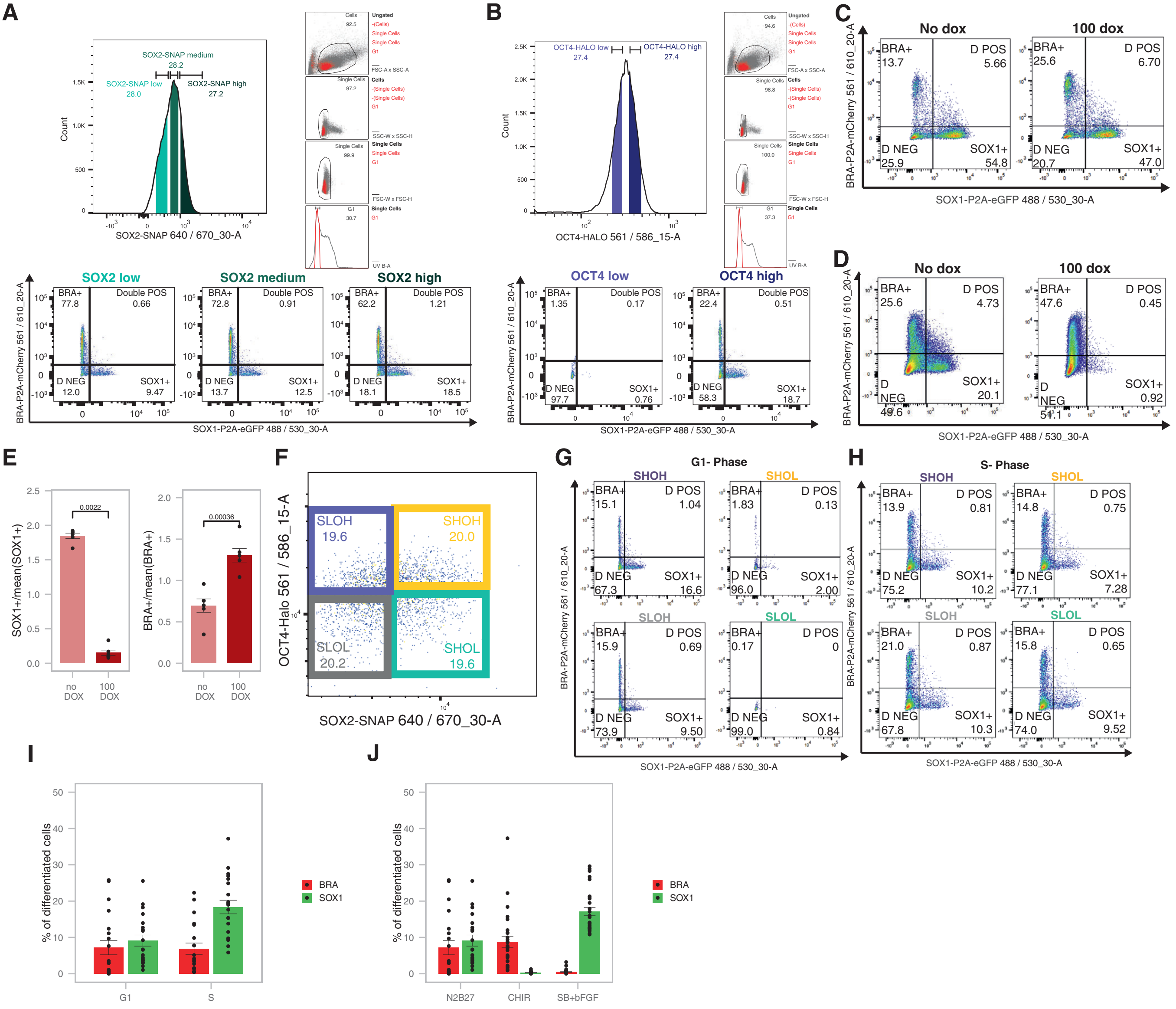

Figure EV4. Impact of SOX2 and OCT4 levels on differentiation outcomes.

A, B Sorting strategies to evaluate the impact of SOX2 (A) or OCT4 (B) levels on differentiation outcomes and example of flow cytometry data after 4 days of differentiation.

C Example of flow cytometry data after 4 days of differentiation with or without OCT4 overexpression for 12 h before differentiation.

D Example of flow cytometry data after 4 days of differentiation with or without dox throughout differentiation.

E Differentiation outcome after 4 days with or without 100 ng/ml dox throughout differentiation. % of $SOX1^+$ and BRA^+ cells are normalized to the average values of each experiment (n = 6). P-values: Mann–Whitney U-test. Error bars: SE.

F Sorting strategy for SHOH, SHOL, SLOH and SLOL populations in G1 phase.

G Example of flow cytometry data of SHOH, SHOL, SLOH and SLOL sorted in G1 phase (see Fig 3I).

H Example of flow cytometry data of SHOH, SHOL, SLOH and SLOL sorted in S phase.

I Percentage of BRA^+ and $SOX1^+$ cells after 4 days of differentiation, when sorted in G1 or S phase (n = 5 biological replicates, including SHOH, SHOL, SLOH and SLOL conditions for each replicate both G1 and S). Error bars: SE.

J Percentage of BRA^+ and $SOX1^+$ cells after 4 days of differentiation in different conditions, when sorted in G1 phase (n = 5 for N2B27, n = 7 for CHIR and n = 7 for SB+bFGF biological replicates including SHOH, SHOL, SLOH and SLOL for each replicate). Error bars: SE.

© 2019 The Authors

Figure EV5. ATAC-seq analysis and characterization of differentially accessible regions.

A Fraction of reads in ATAC-seq peaks for the different cell populations.
B Violin plot of the log2 fold-change in OCT4-high versus OCT4-low and SOX2-high versus SOX2-low in different groups of loci; $n = 3$ biological replicates.
C Metaplots of chromatin accessibility for the different cell populations in different groups of loci; $n = 3$ biological replicates.
D–H Top 12 GO terms of loci in the upregulated SOX2 (D), upregulated OCT4&SOX2 (E), downregulated OCT4 (F), downregulated SOX2 (G), and downregulated OCT4&SOX2 (H) groups.
I Top 12 GO terms for loci in the Upregulated OCT4 group that are bound by OCT4 and with FDR $< 5\%$.
J Percentage of overlap of regions in the different groups with genomic annotations.
K Left: Percentage of regions in the different groups overlapping OCT4 peaks from 4 different datasets (GSE87822, GSE92846, GSE78073 and GSE56138). Right: Heatmaps of OCT4 ChIP-seq signals in the different groups from samples in the 4 datasets (OCT4 untreated (King), OCT4 asynchronous (Liu), OCT4 G1 (Shin) and OCT4 2i (Buecker).
L Fraction of regions in the different groups overlapping OCT and SOX motifs.
M Prediction of group belonging in the test data based on a trained random forest model (see Materials and Methods). The *x*-axis shows the real group belonging, and the *y*-axis shows the percentage of regions predicted to belong to the different regions as coloured in the legend.
N Metaplot of NANOG (GSE87822) binding in the different groups.
O Changes in accessibility of regions upregulated or downregulated in OCT4-high cells upon OCT4 knockdown (GSE87822).

© 2019 The Authors

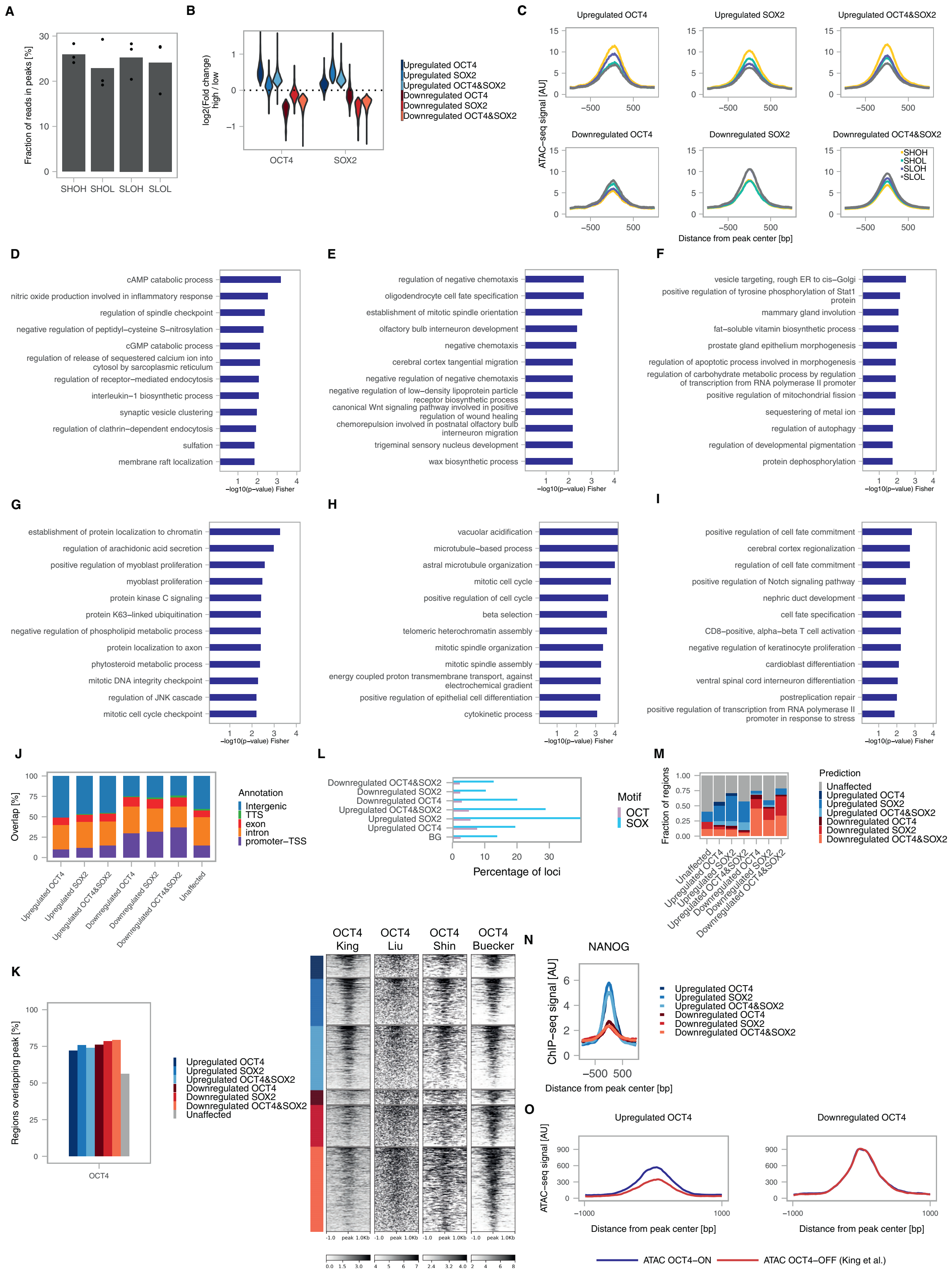

Figure EV5.

 © 2019 The Authors
