# Supplementary material for: Endogenous fluctuations of OCT4 and SOX2 bias pluripotent cell fate decisions
Source: Mol Syst Biol. 2019 Sep 25;15(9):e9002. doi: 10.15252/msb.20199002 (PMC6759502; doi:10.15252/msb.20199002)
Supplement: Supplementary file 16 — Code EV1 [file MSB-15-e9002-s016.zip › Computer Code EV1/README.rtf]

READ MEEndogenous fluctuations of OCT4 and SOX2 bias pluripotent cell fate decisionsStrebinger D, Deluz C, Friman ET,  Govindan S, Alber A, and Suter DMMolecular Systems Biology, 2019* This file describes how to use CellProfiler (version 2.1.1) to analyse mouse ES cell imaging data, as used in the corresponding manuscript. It consists of two different pipelines: 1. “CorrectionFactorExtraction.cpproj”: Pipeline to segment and quantify nuclei from dual Luciferase imaging experiments to calculate a Correction Factor, that can be used to normalise the NanoLuciferase signal and performing quantitative luminescence microscopy. 2. “Analysis_quantitativeIF.cpproj”: Pipeline to segment and quantify nuclear fluorescence signal from immunofluorescence imagesad 1.For these analyses single images from the time-lapse movie are deposited in a dedicated folder (e.g., “Single Images”) and used as input for the analysis pipeline.I. All cell profiler pipelines start by deposing image files in the “Images” Input module.II. Next in the “NamesAndTypes” module, rules are set to assign the different channels to identifiable names (i.e., filenames containing “NLuc” will be called “NLuc”, filenames containing “FLuc” will be called “FLuc”, filenames containing “mask” will be called “mask”)III. There will be four Analysis modules, starting with a module called “IdentifyPrimaryObjects”: In this module we identify imaging artefacts called “CosmicRays” from the FLuc images. The following parameters are used: Input Image: FLucName of primary objects to be identified: CosmicRaysTypical diameter of objects: 1 6Discard objects outside the diameter range?: YesDiscard objects touching the border of the image?: YesThreshold strategy: GlobalThresholding method: MoGApproximate fraction of image covered by objects: 0.01Select the smooting method for thresholding: AutomaticThreshold correction factor: 1.0Lower and upper bounds on threshold: 0.8 1.0Method to distinguish clumped objects: IntensityMethod to draw diving lines between clumped objects: IntensityAutomatically calculate size of smoothing filter for declumping? YesAutomatically calculate minimum allowed distance between local maxima? YesSpeed up by using lower-resolution image to find local maxima? YesRetain outlines of the identified objects? NoFill holes in identified objects? NeverHandling of objects if excessive number of objects identified: ContinueIV. The second analysis module is “IdentifyPrimaryObjects”, which we will use to identify all nuclei in the “mask” image, using the following parameters:Input Image: maskName of primary objects to be identified: bNucleiTypical diameter of objects: 8 30Discard objects outside the diameter range?: YesDiscard objects touching the border of the image?: YesThreshold strategy: GlobalThresholding method: OtsuTwo-class or three-class thresholding? Three classesMinimize the weighted variance or the entropy? Weighted varianceAssign pixels in the middle intensity class to the foreground or the background? ForegroundSelect the smooting method for thresholding: AutomaticThreshold correction factor: 1.0Lower and upper bounds on threshold: 0.3 1.0Method to distinguish clumped objects: IntensityMethod to draw diving lines between clumped objects: IntensityAutomatically calculate size of smoothing filter for declumping? YesAutomatically calculate minimum allowed distance between local maxima? YesSpeed up by using lower-resolution image to find local maxima? YesRetain outlines of the identified objects? NoFill holes in identified objects? After both thresholding and declumpingHandling of objects if excessive number of objects identified: ContinueV. The third module is “MaskObjects”, to remove all nuclei that coincide with cosmic rays. It uses the following parameters:Select objects to be masked: bNucleiName the masked objects: NucleiMask using a region defined by other objects or by binary image: ObjectsSelect the masking object: CosmicRaysInvert the mask: YesHandling of objects that are partially masked: RemoveNumbering of resulting objects: RenumberRetain outlines of the resulting objects? NoVI. The last analysis module is “MeasureObjectIntensity” using the following parameters:Select an image to measure: FLucSelect an image to measure: NLucSelect objects to measure: NucleiVII. Lastly, we want to set the output of the measurements in “View output settings”, to allow the export in Matlab format:Default Output Folder: “Your path” (e.g., /Users/Daniel)Output Filename: “Your output name” (e.g., DefaultOUT.mat)Output file format: MATLABad2. This pipeline allows to segment and measure nuclear fluorescence signal from immunofluorescence images. This pipeline can be used for the quantification of transcription factors, such as “SOX1” in the example pipeline.I. All cell profiler pipelines start by deposing image files in the “Images” Input module.II. Next in the “NamesAndTypes” module, rules are set to assign the different channels to identifiable names (i.e., filenames containing “NLuc” will be called “NLuc”, filenames containing “FLuc” will be called “FLuc”, filenames containing “mask” will be called “mask”)III. There will be three Analysis modules, starting with a module called “IdentifyPrimaryObjects”: In this module we identify the nuclei from fluorescence images. The following parameters are used: Input Image: DNAName of primary objects to be identified: nucTypical diameter of objects: 20 60Discard objects outside the diameter range?: YesDiscard objects touching the border of the image?: YesThreshold strategy: AutomaticMethod to distinguish clumped objects: IntensityMethod to draw diving lines between clumped objects: IntensityAutomatically calculate size of smoothing filter for declumping? YesAutomatically calculate minimum allowed distance between local maxima? YesSpeed up by using lower-resolution image to find local maxima? YesRetain outlines of the identified objects? NoFill holes in identified objects? After both thresholding and declumpingHandling of objects if excessive number of objects identified: ContinueIV. The second analysis module is “EditObjectsManually”, which we will use to correct the segmentation for misidentified objects, using the following parameters:Select the objects to be edited: nucName the edited objects: NucleiAllow overlapping objects: NoRetain outlines of the edited objects? NoNumbering the edited objects: RenumberDisplay a guiding image: YesSelect the guiding image: DNAV. The last analysis module is “MeasureObjectIntensity” using the following parameters:Select an image to measure: DNASelect an image to measure: SOX1Select objects to measure: NucleiVII. Lastly, we want to set the output of the measurements in “View output settings”, to allow the export in Matlab format:Default Output Folder: “Your path” (e.g., /Users/Daniel)Output Filename: “Your output name” (e.g., DefaultOUT.mat)Output file format: MATLAB
